# Uptake, utility and resource requirements of a genetic counselling telephone helpline within the BRCA-DIRECT digital pathway for mainstreamed BRCA testing in patients with breast cancer

**DOI:** 10.1136/jmg-2024-110428

**Published:** 2025-03-12

**Authors:** Bethany Torr, Grace Kavanaugh, Monica Hamill, Christopher Jones, Helena Harder, Sophie Allen, Alice Garrett, Subin Choi, Rosalind Way, Rochelle Gold, Amy Taylor, Rhian Gabe, Anneke Lucassen, Ranjit Manchanda, Angela George, Michael Hubank, Stephen Bremner, Ashu Gandhi, Zoe Kemp, D Gareth Evans, Lesley Fallowfield, Valerie Jenkins, Clare Turnbull

**Affiliations:** 1 Division of Genetics and Epidemiology, Institute of Cancer Research, Sutton, UK; 2 Clinical Trials Unit, Brighton and Sussex Medical School, Brighton, Brighton and Hove, UK; 3 Sussex Health Outcomes, Research & Education in Cancer (SHORE-C), Brighton and Sussex Medical School, Brighton, UK; 4 BRCA Journey, Patient Representative, LEEDS, West Yorkshire, UK; 5 Clinical Genetics, East Anglian Medical Genetics Service, Cambridge, Cambridgeshire, UK; 6 Wolfson Institute of Population Health, London, London, UK; 7 Clinical Ethics, University of Oxford, Oxford, UK; 8 Wolfson Institute of Preventive Medicine, Queen Mary University of London, London, UK; 9 Department of Gynaecological Oncology, Barts Health NHS Trust, London, UK; 10 Gynaecology Unit, Royal Marsden NHS Foundation Trust, London, London, UK; 11 Centre for Molecular Pathology, Institute of Cancer Research Sutton, Sutton, Surrey, UK; 12 Prevent Breast Cancer Centre, Wythenshawe Hospital Manchester Universities Foundation Trust, Wythenshawe, Manchester, UK; 13 Cancer Genetics, Royal Marsden Hospital Sutton, Sutton, UK; 14 Division of Evolution, Infection and Genomic Sciences, The University of Manchester, Manchester, UK

**Keywords:** Genetic Testing, Germline Mutation, Genetic Counselling, Medical Oncology, Health Services Administration

## Abstract

**Background:**

We trialled the first digital pathway (BRCA-DIRECT) aiming to improve capacity for mainstreamed BRCA testing within UK breast oncology services. Patients received standardised digital pretest information, with saliva sampling and consent to testing completed at home. For individualised support, we offered access to a clinical genetics professional via a telephone helpline (TH).

**Methods:**

To evaluate the utilisation, uptake and resource requirements for provision of the TH, we analysed data from structured call logs recorded in the BRCA-DIRECT Study. Mixed-methods analysis included combining quantitative data from call logs and patient demographics with thematic analysis of free-text notes establishing reasons for calls. Additional data were analysed from structured telephone interviews.

**Results:**

Calls were received from 201/1140 (17.6%) patients. We identified that 84.6% of calls (274 calls, 1097 min) pertained to ‘administrative’ support needs only. The remaining 15.4% required a clinical genetics professional (50 calls, 344 min). Of the clinical calls received: 26.0% were placed prior to test consent, 36.0% while awaiting results and 38.0% post results, with median (interquartile) call lengths of 8 (4–10) min; 5.5 (4–10) min; and 5 (3–7) min, respectively. Across all 1140 patients, a mean of 0.3 min of clinical time was required per patient.

**Conclusions:**

Our findings demonstrate that the ‘BRCA-DIRECT’ model of standardised information provision served most patients, with a minority using the helpline for supplementary clinical information or support. The modest per-patient requirement for clinical time supports the scalability of this model for expanding mainstream genetic testing within UK oncology services.

**WHAT IS ALREADY KNOWN ON THIS TOPIC**

- New service delivery models are being trialled within which pretest information and informed patient consent for diagnostic germline genetic testing are provided within oncology settings, rather than by clinical genetics services.
- These pathways are aimed at reducing delays and clinician time required for genetic testing and increasingly involve provision of generic information. Patient support from expert genetics clinicians for addressing individualised questions or psychosocial concerns remains an important element of the process.
- Genetic clinician telephone helplines (THs) have been implemented within several studies for this purpose, but limited data have been published focusing on the uptake, utility and resource requirements for provision of these services.

**WHAT THIS STUDY ADDS**

- Our results reveal that most calls to the TH can be addressed by an informed administrator, pertaining to results turnaround times or support required for accessing or progressing through the digital steps, while minimal clinical genetics specialist time is required on average per patient to address clinical support needs.

**HOW THIS STUDY MIGHT AFFECT RESEARCH, PRACTICE OR POLICY**

- The findings provide additional evidence that models which use standardised information delivery could help to reduce the overall requirement for clinical time involved in diagnostic germline genetic testing pathways.
- However, further research is required to better understand barriers and facilitators to uptake of the TH among patients.

## Introduction

 Diagnostic germline genetic testing at the point of a breast cancer (BC) diagnosis is becoming increasingly relevant. This is due, in part, to the implementation of targeted therapeutics for germline pathogenic variants (gPVs) in *BRCA1* and *BRCA2* (BRCA genes), in particular PARP inhibitors, such as olaparib and talazoparib, for treatment of early BCs and HER-2 negative advanced or metastatic BCs.^1^ Accordingly, eligibility criteria for testing are expanding to maximise the benefit for therapeutic access and demonstrated within England, UK, by the inclusion of new ‘therapeutic’ indications on the National Test Directory (NTD) for Rare and Inherited Diseases, for example, R444 for genetic testing for Olaparib eligibility.^2^ This is in addition to existing indications, aimed at offering testing to those at greater than ~10% likelihood of having a gPV in the BRCA genes, based on BC pathology, ancestry and/or family history criterion.

However, while eligibility criteria are expanding, there continue to be missed opportunities to identify people with gPVs in BRCA genes who may benefit from targeted therapeutic options, as well as interventions for prevention and early detection of future cancers for them and their family. These include risk-reducing bilateral mastectomy for prevention of future primary BCs or risk-reducing salpingo-oophorectomy for prevention of ovarian cancer (OC).^3^ In an analysis conducted in 2023 of unselected BC testing, it was estimated that among women presenting with BC, fewer than 20% were eligible for testing via the NTD criteria, missing approximately half of those identified to have gPVs in high-penetrance BRCA genes (*BRCA1, BRCA2* and *PALB2*).^4^ There is, therefore, a strong argument for expanded access to BRCA gene testing (BRCA testing) among BC cases; current clinical pathways arguably, however, limit our ability to expand our volumes of BRCA testing.

There is a split within the UK context of BRCA testing undertaken at the time of BC diagnosis. In some services, this continues to be delivered predominantly by clinical genetics services following referral by the oncology or surgical team. In many services, however, there has been implementation of ‘mainstreaming’, now widely introduced in OC pathways, where genetic counselling and consent to testing are delivered within the oncology clinic, typically via trained oncology or surgical professionals. Several barriers to implementation of mainstreaming in breast oncology settings have been cited, and consequently, the success in roll-out of mainstreaming observed in OC has not similarly been observed within National Health Service breast oncology services.^5 6^ This disparity in large part reflects the higher volume of BC compared with OC diagnoses per year (~56 000 vs ~7500) with concomitant pressures on clinician time in the breast clinic.^7 8^

Therefore, one way to facilitate expansion of BRCA testing for patients with BC is to reduce clinician time involved in generic aspects of the BRCA testing pathway, for example, by deploying digital delivery of the pretest information traditionally provided as part of genetic counselling or mainstream testing appointments. There have been several previously reported studies of pathways using digital or telemedicine approaches for genetic testing, which have reported reductions in genetic counselling time required per patient and generally high levels of satisfaction. Notably, the context of these studies has typically been community-based testing, for example, of people with a family history of cancer or of Jewish ancestry.^9^,^12^

We designed the first digital pathway (BRCA-DIRECT) for use within breast oncology units for BRCA-gene testing within the UK.^13^ The BRCA-DIRECT pathway was designed to reduce clinical staff time involved in generic aspects of the pathway while maintaining close oversight from, and interaction with, clinical genetics services. Patients were provided with access to an online genetic testing management system (BRCA-DIRECT digital platform). This provided digital information about BRCA testing (pretest information) akin to information usually delivered via a genetic counselling consultation; enabled sharing of information from the patient to the clinician (eg, family history questionnaire/digital consent) and from the clinician to the patient (eg, digital return of negative results); and facilitated booking of timely clinical appointments. Saliva sampling, which could be completed by the patient at home, was also used as the primary method for DNA sampling.

We trialled the pathway via the BRCA-DIRECT Study between 2021 and 2023 for germline *BRCA1*, *BRCA2* and *PALB2* testing in unselected patients with BC. We randomised patients 1:1 to receive either digital pretest information (fully digital pathway) or information within a genetic counselling consultation (partially digital pathway). We demonstrated non-inferiority of digital pretest information in terms of uptake of BRCA testing, knowledge, satisfaction and anxiety.^14^ Overall, satisfaction with the end-to-end digital pathway for both patients and clinicians was high.

One of the central design elements of the BRCA-DIRECT pathway was that the patient has ready access to genetics expertise via a telephone helpline (TH). The hypothesis is that, for most patients, specific questions or individualised support needs can be managed more efficiently via an easily accessible TH (alongside provision of generic information for all patients via a digital pathway), as compared with current standard-of-care one-to-one provision of all relevant information within clinical appointments for all patients. Within the BRCA-DIRECT Study, all patients, regardless of randomisation, had access to the TH. This type of TH service is not currently available as standard of care at the participating sites outside of this study.

Here we present findings, using data from the BRCA-DIRECT Study, on the uptake, utilisation and resource requirements for management of a TH alongside the BRCA-DIRECT digital pathway, implemented within five breast oncology units based within London and Manchester, for diagnostic genetic testing of *BRCA1, BRCA2* and *PALB2* in unselected patients with BC.

## Methods

### Population

The BRCA-DIRECT Study population included 1140 consented patients. Patients eligible for the study comprised: (1) Those with a diagnosis of invasive BC or high-grade ductal carcinoma in situ (DCIS), (2) Female and over the age of 18 years old, (3) Those with access to the internet, and (iv) Those with good comprehension of the English language. Previous BRCA testing was an exclusion criterion.

All patients underwent genetic testing via the BRCA-DIRECT digital pathway, with 569 (49.9%) participants randomised to receive standardised, pretest information digitally (fully digital pathway) and 571 (50.1%) having a genetic counselling consultation (partially digital pathway).

### Intervention

#### TH provision

The TH was available to all clinicians and patients from sites involved in the BRCA-DIRECT Study from 9:00 to 17:00, Monday to Friday. Calls were primarily answered by a Genetic Nurse or Counsellor (GN/C); but could be managed by an administrator who addressed administrative queries or arranged a call-back with a clinical genetics professional (GN/C or Clinical Geneticist).

The availability of the TH was advertised across all stages of the BRCA-DIRECT pathway, advising patients to call if they had questions or required more information and/or support. Details were included within the patient information sheet provided prior to uptake of BRCA testing via the study, on the BRCA-DIRECT platform where patients completed genetic testing and study procedures, and within all written correspondence, including results letters.

### Data collection

#### Patient demographics

Patient demographics (including date of birth, highest qualification, marital status and ethnicity) were patient-provided, via a digital survey within the BRCA-DIRECT platform at baseline. Clinical teams provided the patient’s BC type, treatment stage (newly diagnosed, in follow-up or metastatic), and primary surgical date (if known/applicable).

#### TH call logging

TH calls from patients who had (1) Returned their study consent and (2) Registered to the BRCA-DIRECT platform were captured, by the call handler, in structured call logs, against the patient’s digital record. The following information was captured:

- Date of call,
- Time of call (am or pm),
- Length of call (minutes),
- Call content (free-text notes).

### Outcomes and data analysis

A mixed-methods approach was taken to understand the uptake, utilisation and resource requirements for managing the TH implemented within the BRCA-DIRECT Study.

First, thematic analysis of ‘call content’ captured within call logs was conducted within Excel to distinguish the nature of the TH calls,^15 16^ with data subsequently combined quantitatively with additional patient demographic and study data.

#### Call log inclusion/exclusion

The following call logs were included:

- Call logs capturing any form of contact made to the study team by a patient, or a clinician acting on behalf of the patient.
- Call logs detailing follow-up contact made by study staff or patients to an initial enquiry made by a patient/clinician.

The following call logs were excluded:

- Call logs detailing contact initiated by the study team without prior contact made by a patient or a clinician on the patient’s behalf.

#### Thematic analysis

Anonymous free-text notes were extracted from call logs and independently reviewed by two research GN/Cs within the study team (GK and MHa).

The review first included categorisation of calls into two distinct categories:

- *Clinical:* All or some of the content of calls requiring a clinical genetics professional (GN/C or Clinical Geneticist) to address appropriately.
- *Administrative:* All the content could be satisfactorily answered by an informed administrator and did not require a clinical genetics professional.

Second, the call log content was further analysed for recurring themes under the administrative and clinical categories. Call logs could include more than one instance of a theme.

Categorisation and theme codes were agreed, and subsequently refined by GK and MHa through multiple rounds of analysis and peer debriefing. Any discrepancies between the two coders were reviewed, and, if final consensus could not be reached, were resolved by a third independent reviewer (BT).

#### Quantitative analysis

Following categorisation and thematic analysis of the telephone call logs, the data were combined with additional call log and patient/study data for quantitative data analysis of structured call logs (including breakdowns by the categories and themes) and exploratory comparison of participant demographics between (administrative/clinical) callers and the main study population using binomial probability testing. Analyses were completed using Stata V.17.0.

#### Structured patient interviews

Additional quantitative data were captured separately from structured telephone interviews.

All patients randomised to the fully digital pathway (received digital pretest information) within the BRCA-DIRECT Study were invited to participate in structured interviews on completion of the study pathway (subject to consent for further contact), with 21 participants completing the interviews. Interviews were conducted by GK, MHa and BT.

Within the interviews, a subset of five questions was asked, covering awareness of the TH during the study and capturing the perceived: (1) Necessity of providing the TH and (2) Individual likelihood of using the TH, for both clinical genetics and administrative queries, on a five-point Likert scale.

The participant was asked within the interview to answer the questions based on structured responses (categorical answers or Likert scales). Following completion of the interviews, transcripts were reviewed by two researchers (MHa and BT) to capture additional free-text responses for each of the questions. For this analysis, only quantitative data were analysed descriptively using Stata V.17.0.

#### Sample size

The BRCA-DIRECT Study sample size (1000 patients) was determined to ensure >95% likelihood of identifying at least five individuals with pathogenic variants (PV) from each of the recruiting sites (London and Manchester), based on an estimated PV-detection rate of 2%. Power was not specifically determined for this subanalysis.

## Results

BRCA-DIRECT Study recruitment took place between 05 July 2021 and 15 August 2022, with completion of the study on 16 January 2023. TH calls were logged between the dates of 27 July 2021 to 25 October 2022 (65-week period).

There were 1140 consented patients with BC within the main study. Of these, 667/1138 (58.6%) were newly diagnosed, 400/1138 (35.1%) in follow-up and 71/1138 (6.2%) had metastatic disease; for 2/1140 (0.2%) treatment stage was unknown. The mean age (SD) of the participants was 58.6 (±11.7) years. Most participants were white (84.1%) and educated to degree level or higher (49.9%) (see table 1 and Torr *et al*^14^ for a fuller description of the study demographics).

**Table 1:** Patient demographics. Demographics of patients, split by callers (n=201) and all patients participating in the BRCA-DIRECT Study (n=1140)

Demographic		Callers		BRCA-DIRECT Study participants	
		(n=201)		(n=1140)	
**Age (years**)	Mean, range	58.9 (SD 12.7), 31–89		58.6 (SD 11.7), 30–94	
		**n**	**%**	**n**	**%**
	18–30	0	0.0%	1	0.1%
	31–40	15	7.5%	57	5.0%
	41–50	40	19.9%	243	21.3%
	51–60	54	26.9%	353	31.0%
	61–70	52	25.9%	295	25.9%
	71–80	31	15.4%	155	13.6%
	81 or older	9	4.5%	36	3.2%
	Total with available data	201	100%	1140	100%
**Highest qualification**	No qualification	10	5.3%	71	7.0%
	School level	38	20.0%	221	21.7%
	Post-16 qualification	34	17.9%	218	21.4%
	Degree Level	68	35.8%	330	32.4%
	Higher degree level	40	21.1%	178	17.5%
	Total with available data	190	94.5%	1018	89.3%
	*Missing*	*11*	*5.5%*	*122*	*10.7%*
**Clinical site**	London	147	73.1%	682	59.8%
	Manchester	54	26.9%	458	40.2%
	Total with available data	201	100.0%	1140	100.0%
**Ethnicity**	White	158	80.2%	884	84.1%
	Asian/Asian British	17	8.6%	67	6.4%
	Black African/Caribbean/black British	5	2.5%	35	3.3%
	Multiple/other	17	8.6%	65	6.2%
	Total with available data	197	98.0%	1051	92.2%
	*Missing*	*4*	*2.0%*	*89*	*7.8%*
**Treatment stage**	Newly diagnosed	141	70.1%	667	58.6%
	In follow-up	49	24.4%	400	35.1%
	Metastatic	11	5.5%	71	6.2%
	Total with available data	201	100%	1138	98.8%
	*Missing*	*0*	*0.0%*	*2*	*0.2%*
**Study arm**	Fully digital	90	44.8%	569	49.9%
	Partially digital	111	55.2%	571	50.1%
	Total with available data	201	100%	1140	100%

### Uptake

Out of the 1140 consented BC patients, 201 (17.6%) contacted the TH.

A total of 324 separate calls were logged, with 19/324 (5.86%) being follow-up calls linked to a prior call and 5/324 (1.5%) being made by a clinical healthcare professional on behalf of a patient. Of the 305 primary contacts (ie, excluding follow-up) from the 201 patients, the median (IQR) number of calls was one (one to two), ranging from one to five per patient.

Caller demographics were broadly representative of the study population (table 1). The primary exception to this was distribution by hospital site, where 54/201 (26.9%) callers were from Manchester and 147/201 (73.1%) callers from London, compared with the BRCA-DIRECT Study participants where 458/1140 (40.2%) were from Manchester and 682/1140 (59.8%) from London (p<*0.001*). Additionally, treatment stage was significantly different between callers (141/201 (70.1%) newly diagnosed) and all BRCA-DIRECT Study participants (667/1140 (58.6%) newly diagnosed) (p<*0.001*).

#### Utilisation

50/324 (15.4%) of the calls were categorised as clinical (ie, requiring a clinical genetics professional to address some/all content) and 274/324 (84.6%) as administrative.

Of the unique clinical callers, 14/38 (36.8%) were patients randomised to the fully digital pathway, while 24/38 (63.2%) were randomised to the partially digital pathway. The split was more even for administrative callers, where there were 98/183 (53.6%) in the partially digital pathway and 85/183 (46.4%) in the fully digital pathway.

#### Administrative call content

**Figure 1:**
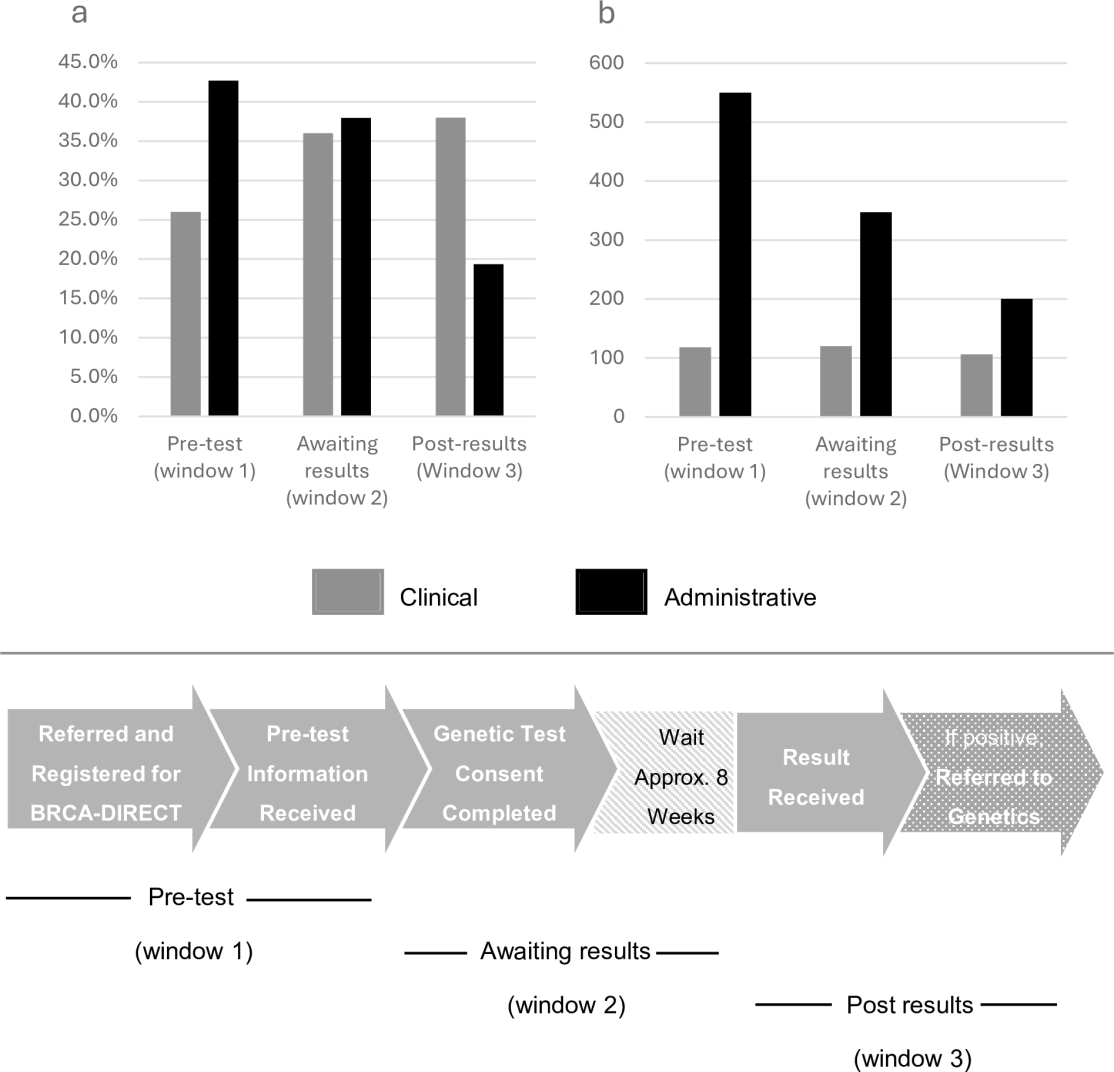
(**a**) Proportion of clinical or administrative calls and (**b**) Total minutes of clinical or administrative calls, received at different time points within the patient pathway: (1) Before the participant consented to genetic testing, (2) While awaiting the results from the testing and (3) After receiving the result from testing.

Of calls categorised as administrative, 221/274 (80.7%) occurred prior to patients receiving their genetic test result (figure 1a).

There were 316 instances of administrative themes arising within 291 calls (including 17 calls which also featured ‘clinical’ call content), ranging from 1 to 2 instances per call. The themes were: access and progress (152/316, 48.1%), results (130/316, 41.1%), samples (14/316, 4.4%), study process and design (9/316, 2.8%), and miscellaneous (11/316, 3.5%) (see table 2, including definitions).

**Table 2:** Themes and subthemes arising within telephone helpline calls. Counts and descriptions of the different themes/subthemes arising within telephone helpline calls classified under broader ‘administrative’ (not requiring a clinical genetics professional) and ‘clinical’ (requiring a clinical genetics professional) categories. Calls could contain more than one instance of a subtheme, each of which is counted independently

Administrative			
**Theme (N**)	**Subtheme**	**n**	**Description**
**DNA sampling (14**)	Saliva sample acquisition	7	Providing and posting a saliva sample
	Blood sample acquisition	2	Providing a blood sample
	Sample failure	5	Sample analysis failure in laboratory
**Access and progress (152**)	Confirming progress	11	Confirming step completed correctly (eg, form submitted)
	Genetic test consent	18	Administrative aspects of completing genetic test consent (eg, digital signature)
	Platform technical issues	71	Registering to or progressing through platform
	Notification received	28	Questions about, or prompted by, an email/SMS notification
	Scheduling telephone appointment	19	Booking or rebooking pretest or results telephone appointment
	Entering or adding family history	4	Administrative aspects of how to enter family history on platform, adding family history post form submission.
	Withdrawal request	1	Request to withdraw from study
**Study process and design (9**)	Research study-specific administrative queries	9	Questions/comments about study protocols, pathway and involvement
**Results administration (130**)	Results turnaround time	111	Inquiring if/when results are available
	Confirming result	15	Confirming negative results after viewing on platform
	Referral to clinical genetics	4	Follow-up regarding onward referral
**Miscellaneous (11**)	Miscellaneous calls	11	Other administrative calls
**Total**		**316**	

Clinical			
**Theme (N**)	**Subtheme**	**n**	**Description**
**Informational needs ahead of consent to testing (19**)	Cancer risk and management	7	Hereditary cancer risks and management options for patient and relatives
	Testing of other cancer genes	3	Testing and risks for genes other than *BRCA1/BRCA2/PALB2*
	Insurance	4	Implications of genetic testing on insurance (eg, life insurance, travel insurance)
	Family history/ancestry	4	Cancer risk based on family history and/or ancestry
	Variants of uncertain significance	1	Variants of uncertain significance
**Psychosocial needs (20**)	General support regarding testing/process	8	Support around decision making, family dynamics, coping strategies, etc.
	Anxiety related to test turnaround time	12	Anxiety while awaiting test results
**Further questions on negative results (13**)	Family members (risks, screening, testing)	8	Hereditary cancer risks and management options for relatives
	Testing of other cancer genes	2	Testing and risks for genes other than *BRCA1/BRCA2/PALB2*
	Variants of uncertain significance	1	Variants of uncertain significance
	Miscellaneous	2	Other clinical inquiries
**Further questions on positive results (2**)	Further questions following a positive result	2	Questions relating to receiving a positive result and downstream referrals
**Study process and design (5**)	Research study-specific clinical queries	5	Questions/comments about study protocols, pathway and involvement
**Total**		**59**	

The theme of ‘access and progress’, representing the major administrative theme arising in calls (152/316, 48.1%), encompassed different aspects of interacting with the BRCA-DIRECT platform, including the following subthemes: ‘Portal technical issues’ (71/152, 46.7%), ‘responding to notification’ (28/152, 18.4%) and ‘scheduling a telephone appointment’ (19/152, 12.5%).

However, the most common administrative subtheme arising, captured under the theme of ‘results’, was ‘result turnaround time’ (111/316, 35.1%).

#### Clinical call content

There were 59 instances of themes arising within the 50 calls categorised as clinical. These themes included: informational needs upfront of testing (19/59); psychosocial needs (20/59); further questions on receiving negative results (13/59); further questions on receiving positive results (2/59); and study process and design (5/59).

Of calls categorised as clinical, only 13/50 (26.0%) were recorded prior to patients consenting to testing (figure 1a). 3/13 of these were from patients who received their ‘pre-test information’ digitally and 10/13 from patients within the partially digital pathway (ie, had a pretest telephone consultation with a genetic counsellor), representing 0.5% and 1.8% of patients randomised to the respective arms.

There were 19 instances of the ‘informational needs upfront of testing’ theme occurring within the 13 clinical calls recorded prior to patients consenting to testing. This ranged from one to three instances per call. Subthemes arising under this theme were: general cancer risks and management options (7/19, 36.8%), cancer risks based on family history/ancestry (4/19, 21.1%), insurance (4/19, 21.1%), other genes beyond *BRCA1/BRCA2/PALB2* (3/19, 15.8%) and variants of uncertain significance (1/19, 5.3%).

Of clinical calls, 18/50 (36.0%) were recorded from patients awaiting results. Within 12 of the calls received at this time, arose the theme of ‘psychosocial needs’ specifically pertaining to ‘anxiety related to test turnaround time’. This was the most common clinical subtheme arising, reflecting ‘turnaround time’ also being a common administrative subtheme.

Overall, the greatest proportion of clinical calls were recorded after results had been received (19/50, 38.0%). Two calls were recorded from people with further questions on receiving positive results, representing 2/30 (6.7%) of those with a positive result, compared with the 13/969 (1.3%) people contacting the TH with ‘Further questions on receiving negative results’. Of these, questions mostly pertained to information for family members (8/13, 61.5%).

#### Patient feedback

**Figure 2:**
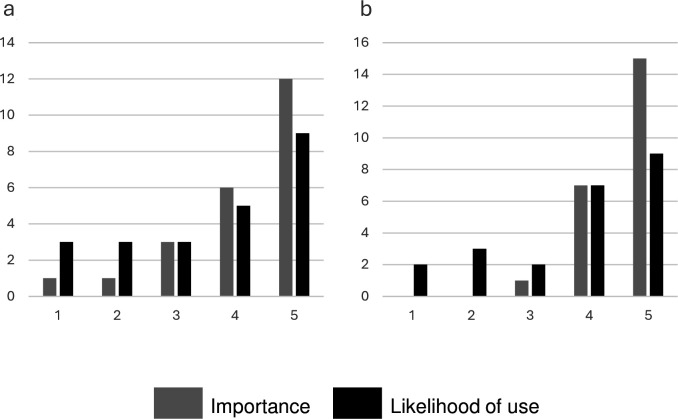
Patient-reported importance of having the telephone helpline (grey) and likelihood of use (black) for (**a**) Administrative support or (**b**) Clinical genetics support. Questions were scored on a 5-point Likert Scale: 1—very unimportant/likely; 2—somewhat unimportant/likely; 3—don’t know/depends; 4—somewhat important/likely; 5—very important/likely, based on structured interviews with 21 patients randomised to receive digital pretest information (fully digital arm), of whom 2/21 (9.5%) reported using the telephone helpline.

Of the patients undertaking structured interviews, 16/21 (69.6%) reported they were aware of the TH and 2/21 (9.5%) reported using it for clinical support. Most respondents expressed they felt it to be either ‘somewhat’ or ‘very’ *important* to provide a TH alongside a digital pathway for clinical support (20/21, 95.2%) and/or for administrative support (18/21, 85.7%) (figure 2). However, only 16/21 reported they themselves would be ‘somewhat’ or ‘very’ *likely to use* the TH for administrative or clinical support if they had questions, with 4/21 (19.0%) and 3/21 (14.3%) reporting they were ‘somewhat’ or ‘very’ *unlikely to use* the TH for respective support.

### Resource requirements

In total, 1441 min of time was recorded against the 324 TH calls (clinical: 344 min; administrative: 1097). Clinical calls ranged from 2 min to 28 min, with median (IQR) call lengths of 5 (4–10) min. By contrast, administrative calls ranged from 1 min to 20 min per call, with a median (IQR) call length of 3 (2–4) min.

In consideration of costing the resource required for administering the TH: across all 1140 consenting patients, the average (mean) minutes of call time was 1.3 min per patient (consisting of 0.3 min for clinical calls and 1.0 min for administrative calls).

When considering the time point in which clinical calls were received, the 26.0% of calls received prior to genetic test consent totalled 118 min, with a median (IQR) call length of 8 (4–10) min. After this stage, the median (IQR) call lengths were lower (post-test consent, awaiting results: 5.5 mins (4–10); post receiving results: 5 mins (3–7)), however, the total time required overall was similar (post consent, awaiting results: 120 min; post receiving results: 106 min) reflecting the higher volume of calls during these windows (figure 1b).

## Discussion

Overall, findings indicate that for mainstreamed BRCA testing of patients with BC, provision of a TH alongside a digital pathway with standardised pretest information is feasible, given the modest clinical time required for addressing calls. Requirement for GN/C support was found to be limited prior to BRCA testing consent, with an increase in total time observed downstream for calls from patients who had received their results. There may, however, be issues with patient awareness/recollection regarding the existence of the TH, as well as hesitancy towards patients contacting the TH, which require further exploration.

### Majority of the calls to the TH were administrative

The majority of the calls were administrative (84.6%), that is, all of the call content could be addressed by an informed administrator and did not require a clinical genetics professional, with themes arising within administrative calls highlighting both the direct and indirect challenges with implementation of a digital pathway.

Regarding the direct challenges of the digital pathway, the most common theme arising from administrative calls was ‘access and progress’ while interacting with the BRCA-DIRECT digital platform. This activity is thus likely to be in excess to the support that would necessarily be required within a standard-of-care, clinic appointment-based pathway where a digital platform is not routinely implemented.

Regarding the indirect challenges of a digital pathway, ‘testing turnaround times’ was the most common subtheme arising. Specifically, during the study period, delays to test turnaround times were caused due to unforeseen COVID-19 impacts on staffing. With the turnaround time advertised to patient within the BRCA-DIRECT platform being difficult to update dynamically, this highlighted an indirect challenge associated with implementing ‘standardised’ information via a digital system that resulted in high call volumes.

These observed themes, therefore, demonstrate that many of the administrative reasons for calls are highly likely to vary by the specifics of the pathway (eg, complexity of accessing the digital platform or variability of turnaround time).

Nonetheless, despite accounting for the majority of calls, the time requirement to address administrative calls when averaged across all 1140 participants remained low (1.0 min per patient). Thus, while within the study we took a ‘GN/C-first’ approach to answering the TH, findings strongly support an administrator-first model to address the bulk of calls, with option for triage to a clinical genetics professional as required.

### THs as a feasible supplement to standardised pretest information

For clinical calls (ie, those requiring a clinical genetics professional), the overall amount of time required per patient was also low (less than 1 min when averaged across all 1140 patients). Findings also revealed that the proportion of patients contacting the TH requiring additional clinical information or counselling ahead of consenting to BRCA testing was low (1.1%); notably this was lower in the group randomised to receive digital pretest information (0.5%) compared with those patients having a pretest genetic counselling consultation (1.8%).

This supports one of the main hypotheses of the BRCA-DIRECT Study, that provision of high-quality, well-designed generic pretest information resources satisfactorily addresses, for most patients, their information needs and questions prior to consenting to BRCA testing, with limited additional clinical time required to address the needs of those with extra questions or individualised counselling needs.

### Readily available genetics support is important for patients following the receipt of BRCA test results

It is important to note, however, that there was a higher rate observed for calls to the TH requiring a GN/C following patients receiving their results. Proportionately, TH usage was higher for those receiving a positive result (6.7% compared with 1.3% for a negative result), although a greater number of calls were from those with a negative result (given this accounts for 97% of results). Of note, all positive result disclosures were by telephone with a GN/C; logged TH calls were following this appointment.

This demonstrates the importance of access to clinical genetics support for patients receiving (1) Positive results, for whom referral or attendance to clinical genetics may be missed following mainstream testing as highlighted by previous studies^17^,^19^ and (2) Those with negative results who, typically following mainstreamed BRCA testing, would only be seen by healthcare professionals from oncology/surgical specialities. Indeed, individualised genetics expertise following a negative result is likely to be a particularly valuable offering from the TH for patients concerned about residual risks for themselves and family members. Negative results are increasingly returned by letter from genetic testing undertaken within Clinical Genetics’ services; it may be that a sizeable proportion of these might also use a TH if available for additional individualised clinical information on personal and familial risk.

### Support may be sought by patients from oncology or surgical teams

Several contacts to the TH were by the treating oncology and clinical teams, who contacted the TH on the patient’s behalf for either clinical or administrative support. These call logs were included in this analysis as thematic review revealed the calls were triggered specifically by the patient, for example, when physically present in clinic with their clinical team. Demonstrating an additional potential benefit of the TH in providing a quick-access conduit between patients, BC clinicians and clinical genetics within the mainstreaming pathway. This was specifically observed in relation to patients contacting the TH anxious about their testing turnaround time in the context of timely surgical decision-making, the complexities to which have been identified as a common challenge for surgical teams.^5^

### Awareness of and hesitancy in patients contacting the TH

Interviews with patients revealed that a considerable proportion (~30%) were unaware of the TH despite this being advertised throughout the study. This may reflect recall at the point of conducting the interviews or could be a result of patients not proactively seeking information or acknowledging messaging as they themselves had not required additional support. Nevertheless, awareness of the TH may have been a factor influencing uptake of the TH, as well as efficacy of the service.

Patients involved in interviews also indicated a degree of hesitancy in using the TH themselves (4/21 and 3/21 reporting they were ‘somewhat’ or ‘very’ unlikely to use the TH for administrative or clinical questions, respectively), despite being supportive of the need for the TH. The specific reasons why these individuals reported that they thought it unlikely they would use the helpline were not further explored: this may reflect some hesitancy around accessing a helpline or may just be an articulation from these individuals that they found the default administrative/clinical provision to be sufficient.

Notably there was a lower uptake of the TH for people in the fully digital arm, who had not been required to speak with a GN/C, indicating that requirement to speak with a GN/C for pretest information may have lowered resistance to subsequent accessing of the TH. This could arguably, in some part, reflect differences in whether patients felt they received sufficient information from the digital information compared with those having a GN/C consultation. However, in the main study at final follow-up (28-days post receiving results), patients were asked how they found the amount of pretest information, and >90% in both arms said this was ‘about right’, with no significant difference observed between the groups (unpublished data). For many patients with BC under active follow-up, there is an established relationship with their treating team, particularly the Clinical Nurse Specialists who act as patient advocates, which may thus reduce the requirement for support or information compared with community-based genetic testing.

### Barriers to uptake in different patient groups

To identify whether there were barriers faced by specific patient groups, we performed a comparison of the demographics of callers to those of the full study population. Notably, we observed no difference in TH uptake proportionately by age, highest qualification, ethnicity or study arm. We observed a modestly higher TH usage in newly diagnosed patients (70.1%) compared with patients in follow-up (24.4%); this largely reflected the higher TH uptake from London patients (in which the proportion of ‘newly diagnosed’ cases was commensurately higher) compared with Manchester patients.

### Limitations of this study

This study was not specifically powered for analysis of TH uptake by timing, different demographic groups or according to theme/subtheme. Some findings were also based on interviews with a small subsection of 21 patients from the full study cohort (such as awareness of the TH) which provide an insight into patient experience and preferences but are not more generalisable.

It is also important to consider the implications of this study population comprising unselected patients with BC, as compared with current eligibility restrictions for standard-of-care BRCA testing. In standard-of-care testing under current eligibility criteria, the pick-up rate of those with positive results will be approximately 10% (compared with 3% within the study), with those eligible for testing having a stronger family history of relevant cancers. These factors could potentially lead to greater proportionate use of the TH. Additionally, patients more likely to face barriers in line with recognised health inequalities, such as those without access to internet or comprehension of English, were excluded from the study.

Furthermore, there is an inherent selection bias insofar as the patients accessing the TH were those who felt least hesitant; consequently the themes/subthemes of concern from more hesitant patients are less likely to be captured. More research is therefore required to fully explore the barriers and facilitators to uptake of the TH and understand implications for people who choose not to, or face barriers to, contacting the TH.

Finally, there were calls upstream of consent to the research study, specifically when the patient received their saliva sample kit and study information sheet, that were not recorded systematically and therefore could not be captured within this analysis, owing to structured call logs needing to be specifically associated with a BRCA-DIRECT patient record (ie, those who had consented to the study and were registered). Anecdotally, these upstream calls also largely pertained to administrative aspects of the pathway, either specific to saliva sampling or participation in the study. Nevertheless, the exclusion of these calls from the analysis is a limitation, with both, the themes arising and resource requirements, not accounted for.

### Contextualising findings with previous research

Limited studies have been reported to date, looking at THs in the context of hospital-based genetic testing services for (cancer) patients. A study from 2008 indicated that, in the context of *BRCA1* testing, women felt it to be of value to have access to a TH after provision of written educational information, a finding recapitulated in the patient surveys conducted within our study. A more recent study by Gaba *et al* reported on uptake and utilisation of a TH implemented alongside a digital decision aid for patients with OC. Findings were similar, with only 12.6% of patients contacting the TH and the majority of calls received (75.0%) relating to administrative aspects of the ‘study’.^20^ However, the sample size in this study was small (13 callers out of 103 patients compared with 201 callers out of 1140 patients within our study). Our study is also the first, to our knowledge, to report on the delivery of a TH to support a digital pathway for BRCA testing within the context of a BC mainstream setting.

## Conclusions

We have identified the core themes of calls, highlighting the areas and timepoints where additional information and support from both a clinical and administrative perspective may be required by 1140 unselected patients with BC undertaking mainstreamed BRCA testing. We have quantified and demonstrated as overall modest, the usage volume of the clinical helpline time required per patient with BC undertaking BRCA testing. This supports the TH model potentially facilitating expansion of BRCA testing in the face of limited clinical professional resources. While further research is required to understand better the barriers and facilitators to uptake of the TH, patient surveys demonstrate strong support for provision of a TH alongside a digital BRCA testing pathway. Overall, this study offers further support for the scalability of a digital model for BRCA testing, where standardised information is supplemented by access to a TH staffed by clinical genetics professionals.

## Data Availability

Data are available upon reasonable request.
